# Implementation of Incident Learning in the Safety and Quality Management of Radiotherapy: The Primary Experience in a New Established Program with Advanced Technology

**DOI:** 10.1155/2014/392596

**Published:** 2014-07-22

**Authors:** Ruijie Yang, Junjie Wang, Xile Zhang, Haitao Sun, Yang Gao, Lu Liu, Lei Lin

**Affiliations:** Department of Radiation Oncology, Peking University Third Hospital, Beijing 100191, China

## Abstract

*Objective*. To explore the implementation of incident learning for quality management of radiotherapy in a new established radiotherapy program. *Materials and Methods*. With reference to the consensus recommendations by American Association of Physicist in Medicine, an incident learning system was specifically established for reporting, investigating, and learning of individual incidents. The incidents that occurred in external beam radiotherapy from February, 2012, to February, 2014, were reported. *Results*. A total of 28 near misses and 5 incidents were reported. Among them, 5 originated in imaging for planning, 25 in planning, and 1 in plan transfer, commissioning, and delivery, respectively. One near miss/incident was classified as wrong patient, 7 wrong sites, 6 wrong laterality, and 5 wrong dose. Five reported incidents were all classified as grade 1/2 of dosimetric severity, 1 as grade 0, and the other 4 as grade 1 of medical severity. For the causes/contributory factors, negligence, policy not followed, and inadequate training contributed to 19, 15, and 12 near misses/incidents, respectively. The average incident rate per 100 patients treated was 0.4. *Conclusion*. Effective implementation of incident learning can reduce the occurrence of near misses/incidents and enhance the culture of safety.

## 1. Introduction

With the rapid development of equipment and technology, the technique of radiotherapy becomes more and more complicated. The treatment planning and delivery of radiotherapy are a highly complex, multiple-step process, with multiple professional groups involved. Every step of the care process in radiation oncology requires knowledge in the management of cancer and certain benign disease, radiobiology, medical physics, and radiation safety that can only be achieved by systematic and structured training. There are many steps where incidents might occur, although major radiotherapy incidents are rare. And it is challenging to determine the actual error rate in radiotherapy. The quality management of radiotherapy is paramountly important to guarantee the appropriateness, quality, and safety of radiotherapy.

As a systematic tool and approach of quality management, incident learning has proved its value in many industries [1]. It has also already been successfully used for a number of years in several radiation oncology clinics. A voluntary international reporting system (Radiation Oncology Safety Information System (ROSIS)) has been in online use for 9 years [2]. The International Atomic Energy Agency (IAEA) is leading the development of a multi-institution incident learning system, called Safety in Radiation Oncology, SAFRON. American Society for Radiation Oncology (ASTRO) has called for a national incident reporting/learning system as part of its six-point “target safely” plan to improve patient safety in radiotherapy. The American association of physicist in medicine (AAPM) Work Group on the Prevention of Errors (WGPE) published the consensus recommendations for incident learning database structures in radiation oncology in 2012 [3]. Learning radiotherapy incidents or near misses through incident reporting systems may improve the patient safety and clinical service quality [4]. Reporting, acting on, and learning incidents and near misses may also provide assessment of the success or failure of the quality assurance program in preventing error. Sharing knowledge about incidents allows better process optimization with information about likely severity and frequency of specific errors and helps prioritize quality management initiatives [5–8]. A number of references suggest reporting and learning radiotherapy incidents or near misses.

While it is generally accepted that incident learning is important in the radiation therapy setting, implementing incident reporting and learning represents a considerable challenge. It requires additional clinical resources and a well-designed system for report, analysis, and response. What is more important is the development of a more open mindset and just culture for reporting near misses and incidents, with an increased emphasis on incident learning to uncover latent error pathways. Studies analyzing the effects of incident learning on patient safety and quality in individual clinics remain scarce, while several departments reported their experience [9–13].

The purpose of this work is to explore the implementation and effectiveness of incident learning using a system developed based on AAPM recommended database structure in a new established program of radiation oncology with advanced technology.

## 2. Materials and Methods

### 2.1. Radiotherapy Program Description

Our department of radiation oncology is a comprehensive clinical and academic department with new established external beam radiotherapy program, delivering 537 treatment courses on Trilogy (Varian, Palo Alto, CA) in 2012 and 725 treatment courses on Trilogy and Axesse (Elekta AB, Stockholm, Sweden) together in 2013. Trilogy and Axesse were put into clinical use after commissioning on February 2012 and April 2013, respectively. The comprehensive record-and-verify/information system of Aria (Varian, Palo Alto, CA) and Mosaiq (Elekta AB, Stockholm, Sweden) were used for Trilogy and Axesse, respectively. All the simulation, planning, delivery, and information systems were newly installed and commissioned. All the protocols and quality management programs were established from scratch. For simulation, Brilliance Big Bore CT (Philips Medical Systems, Cleveland, USA) was used. Eclipse and Oncentra (Version 3.2.3; Elekta/Nucletron, Veenendaal, Netherlands) treatment planning systems were mainly used for planning, in addition to Pinnacle (Philips Radiation Oncology Systems, Fitchburg, WI) and Monaco systems. Most of the staff had no previous intensity-modulated radiation therapy (IMRT) and image-guided radiation therapy (IGRT) experience. The techniques utilized included three-dimensional radiotherapy (3D-CRT), IMRT, IGRT, volumetric modulated arc therapy (VMAT), gated treatment, ultrasound guided radiotherapy, four-dimensional (4D) cone beam computed tomography (CT), and Hexapod (Schwabmünchen, Germany) treatment couch with six degrees of freedom. The plans were transferred electronically to the record and verify system by Digital Imaging and Communications in Medicine (DICOM). All the plans were checked by senior physicist after radiation oncologist approval, before treatment. At treatment, the therapist rechecked the transferred data with printed plan data and treatment chart parameters.

### 2.2. Development of the Reporting and Analysis System

With reference to the consensus recommendations for incident learning database structures in radiation oncology by American Association of Physicist in Medicine (AAPM), an incident learning system was specifically designed and developed for reporting, investigating, and learning of individual external beam radiotherapy incidents in our department. The definitions, process maps, severity scales, causality taxonomy, and data elements were referred from AAPM recommendation. The definitions of terms were clarified. 91 common steps were identified for external beam radiation therapy, 35 safety barriers were set. A ten-level dosimetric and medical severity scale were used to reflect the observed or estimated harm to a patient and a radiation oncology-specific root causes table was used to facilitate the root-cause analyses. Near-miss incidents were assigned with the estimated harm that would have occurred had the incident reached the patient.

### 2.3. Clarification of Definitions

We applied the definitions of terms in accordance with the consensus recommendations American Association of Physicist in Medicine (AAPM) [3]. An incident was defined as any unwanted or unexpected change from a normal system behavior which causes or has the potential to cause an adverse effect to persons or equipment. A near miss was defined as any event or situation that could have resulted in an accident, injury, or illness but did not either by chance or through timely intervention. We reported both incident and near miss mainly to highlight the fact that all deviations are potentially of interest even those that do not necessarily impact the patient. Safety barrier was defined as any process step whose primary function is to prevent an error or mistake from occurring or propagating through the radiotherapy workflow.

### 2.4. Clinical Implementation

After pilot testing, clinical staff used the system for reporting the incidents that occurred in clinical practice. The incidents reported in the system were then investigated, analyzed, and learned by a multidisciplinary team which includes the leaders of radiation oncology, radiation physics, and technologists. The priority of our implementation strategy was to strenuously promote staff acceptance and participation through emphasis of senior management participation and expectations with respect to the establishment of a just environment. It was also to promote openness about reporting incident, emphasis on learning and support, and an obligation to act, where all staff members felt comfortable disclosing incidents while maintaining professional accountability. The established multidisciplinary team directs the reporting, investigation process and determines the corrective actions and follow-up, as required to ensuring prompt response and feedback.

The reporting process starts with the initial reporter who submits the initial description of the event. The event is then forwarded to supervisors of the involved areas by the team leader (the chief of the department). In our implementation, even though there may be several areas involved in the event, each event is processed by only one supervisor who performs the initial analysis of the event and determines the best approach for corrective action. Which supervisor is selected to address an event depends on the main area of involvement and on which supervisor believes to be in the best position to address the issue. Each supervisor can review other supervisor's comments and event disposition. This transparency and active involvement of supervisors in event processing contributed significantly to the uniformity of event processing by different supervisors.

The investigation of the incidents was addressed individually by a small number of key supervisors. A full team investigation is used for the more serious incidents and for the incidents where the determination for the basic cause and identification for the most appropriate learning component requires more rigorous assessment. Immediately after reporting or monthly all reported incidents were reviewed at the department multidisciplinary quality management forums. As we collect a broad spectrum of events, from minor near misses to potentially serious incidents, the event processing can vary significantly from one event to another, and this process evolved over time as our clinic gained experience with the system. Low severity events that have already been addressed and at first do not appear to require systematic process changes, such as minor work flow variation or deviation near misses, are typically only classified and cataloged so that their statistics can be reviewed at a later time. Events that are indicative of process weaknesses or the need for training are immediately addressed by the supervisor, who initiates procedural changes or schedules the necessary training. These events are typically judged to be essential for the system operation and corrective actions are relatively simple. The event severity is reviewed and assigned by the supervisor during the investigation and review of the event. Events are subsequently processed based on the severity classification. More complex events that require significant corrective actions are forwarded to the department Process Improvement Committee for further discussion and processing.

All relevant cause/contributory factors were investigated and assigned by the investigating team, from the aspect of management, technique, personnel, equipment, and process, since it is a rare situation in which only one single cause is at work, so that a full root-cause analysis can be implemented. In addition, each discipline was further discussed or reflected on the data within their own specialty, focusing on process breaks and system errors, intent to find means of preventing reoccurrence. Departmental no blame policy was advertised repeatedly encouraging staff to report incidents without fear of retribution. Blaming an individual or group as the only root cause may lead to corrective actions that fail to address an underlying cause. An individual's actions must be viewed in the context in which they act when performing root-cause analysis. Each reporter can know about all events that they have submitted along with the analysis performed by the supervisor and the ultimate disposition of the events.

Learning actions included focused education sessions on a specific process to highlight error risk, process mapping, protocol changes, quality control measures and approaches of prevention of errors and continuous improvement, discussion with specific workgroups to make them aware of any error risk that has been identified, and presentations at chart rounds. The incident learning was used for guidance in reengineering clinical processes. The data and the statistics collected through the established database were presented at the department quality improvement conference with the corrective actions, thus allowing easy communication of problem details and corresponding process modifications.

The reported external beam radiotherapy near misses and incidents were presented from February 2012 to February 2014. The reporting trends for the second year were compared to the events that were reported in the first year.

## 3. Results

A total of thirty-three reports were analyzed, including 28 near misses and 5 actual incidents. During this period, a total of 1262 patients were treated. The summary of reported near misses and incidents are given in Table 1. The average incident rate per 100 patients treated was 0.4; this rate fell to 0.28% in the second year from 0.56% in the first year. The rate of near miss fell to 1.24% from 2.22%.

Table 2 gives the incident category of reported error type. The rates of all types of incidents/near miss decreased except the suboptimal plan quality type. Among them, the wrong patient incident occurred due to the wrong patient plan calling out with the same last name of two patients, which was found when the other patient was treated in the same afternoon. Of the 7 wrong site near misses/incidents, 2 occurred due to the field shape unchanged to conform to the target projection after adding wedge and adjusting the collimator angle, not noticed by the planner, found and corrected during the plan physics review. Three incorrect shifts from computed tomography (CT) reference marks were reported, due to the wrong/missing setting the fiducial reference point when planning, which were caught during physics check and CT repositioning before treatment. One geographic miss resulted from forgetting shifting the treatment isocenter after modifying plan with the prescription dose delivered to the wrong volume. One electronic transfer error of treatment parameters from the treatment planning system was reported.

Of the 6 wrong laterality near misses/incidents, 4 occurred due to the contouring and planning at the wrong (healthy) side of the body, 2 due to wrong patient position selected in the CT scan protocol (head first supine, instead of foot first supine), which were found during the image guided verification before treatment.

Of the 7 dose prescription near misses/incidents, 2 near misses occurred due to the erroneous planning instructions in the prescription, 1 inconsistent prescription in terms of total dose, dose per fraction, and fractions (PTV 50.40 Gy/1.8 Gy/25 f). The other 4 occurred due to planner not following the physician's prescriptions, including significant error of prescription of 60.00 Gy/2.0 Gy/30 f, plan with 60.00 Gy/0.3 Gy/200 f.

Of the 5 wrong dose near misses/incidents, 1 dose calculation error occurred due to the misunderstanding of source axis distance (SAD) factor for manual dose calculation by the junior physicist, which was found and corrected during physics review. The second near miss was an error in absolute dose calibration of electron beam during commissioning of Trilogy which would result in approximately 60 patients treated with a dose 5% below that prescribed per year if not found. This near miss was caused by wrong understanding of the calibration protocol, found, and corrected in physics review. As the cause was a single error, this incident was categorized as one incident despite the impact on a large number of patients if not detected. The other reasons of wrong dose error included insufficient scan field of view, not enough air flash in planning.

The reasons of the 7 reported suboptimal plan quality near misses/incidents included incomplete contouring of organs at risk, not optimal in selection of beam energy, margin setup, intermediate, and low dose distribution.

The incident breakdown by origin is shown in Table 3. The proportion of incidents is highest at the planning stage. One near miss and two incidents occurred in the safety barrier of the radiotherapy process, including one incident in verification of patient ID before treatment delivery, one incident in image-guided verification (we used CT simulator for couch shift and isocenter determination in our department.) after plan modification, and one near miss in commissioning for electron dose calibration.

Of the 33 incidents and near misses reported, 5 originated in imaging for planning, including error/miss marking reference point on patient in software for three patients, error in input patient position (head first instead of foot first) in CT scanner during CT image acquisition for two patients. Of the 25 incidents/near misses originated in treatment planning, seven originated in the preliminary prescription parameters, including 3 wrong prescriptions from oncologist and 4 due to planners not following physician's prescription in terms of fractions, dose per fraction, and total dose. 11 near misses/incidents originated in dose distribution optimization leading to suboptimal plan. The other near misses/incidents included 4 due to targets contoured and planned on the wrong side (healthy side), 1 organ at risk (spinal cord) incompletely contoured leading to overdose if delivered, 1 treatment plan parameter transfer error due to the network issue, and 1 manual dose calculation error for a patient simulated with conventional X-ray simulator, due to the wrong understanding of SAD factor.

The dosimetric and medical severity distribution of reported near misses and incidents are given in Tables 4 and 5. Five reported incidents were all classified as grade 1/2 of dosimetric severity, 1 as grade 0, and the other 4 as grade 1 of medical severity.

The summary of the root causes and contributory factors to the near misses/incidents are given in Table 6. Among them, negligence, policy not followed, training and failure to develop an effective plan, and communication contributed to 19, 15, 12, 5, and 3 near misses/incidents, respectively.

## 4. Discussion

Our results demonstrated that incident learning can be used for the safety and quality management of radiotherapy, even for a department with new established program with advanced technology, new equipment from different vendors, no much safety, and quality management experience. Implementation of an effective incident learning system may serve to reduce the occurrence of actual incidents and enhance the culture of safety at the individual health care professional level and at the multidisciplinary team level by addressing quality improvement initiatives collaboratively with transparent accountability. Incident learning also improved event communication and identification of clinical areas which needed process and safety improvements and encouraged the reporting of potential incidents as a proactive means of enhancing safety and quality in a radiation treatment program. The reported data were also useful for the evaluation of corrective measures and recognition of ineffective measures and efforts.

Implementing Incident learning in radiotherapy is a systematic and complicated project. A rigorous system of learning, feedback, and action are required for this approach to have a meaningful impact on patient care. The corresponding departmental infrastructure and facilities, organization, and culture are needed. The related academic society, organization, and state health administrative department should encourage and protect the reporting and learning of near misses/incidents by advocacy, regulation, and legislature. Step by step improvements are needed for incident learning fully implemented in radiotherapy.

AAPM recommended database structure provided a very good reference for establishing individualized database for individual institution. A reporting system easy to use, file, report, respond, and analyse could be established based on that considering of the context of individual department. The individual events could be quickly and easily reported without disrupting clinical work. The specially designed incident reporting systems in a radiotherapy setting can provide valuable data for process and patient safety improvement. There are some differences in the work process map and quality control program for different institutions, such as using CT simulator for couch shift and isocenter determination before treatment in our department, in which 7 near misses were found and corrected. There is no this process in the AAPM recommended database structure. Only one near miss and two incidents occurred in the safety barrier of the radiotherapy process of the 33 reported near misses/incidents. Erroneous prescription instructions and failure to follow the physician's prescriptions lead to 7 dose prescription near misses/incidents, but no safety barriers were set from prescription and planning to plan review whether for physician, planner, or physicist. Only physics review was set as safety barrier in the AAPM recommended structure. So, the setting of safety barrier should be catered to the individual context of the department. Safety barriers should be added before plan review to reduce errors and delay and improve efficiency. Cox et al. [14] found modification of 36% contouring or prescription in the round after several treatment fractions. So, they suggest that the contouring and prescription review should be completed before planning. Although Ford et al. [15] found that physics review was the most effective safety barrier before treatment delivery, which can find approximately 60% errors, image-guided verification before treatment delivery is another important safety barrier, which found 7 near misses/incidents in our study.

In addition, the severity assignment of an actual incident or near miss is difficult, especially for the near-misses since one has to estimate the harm that would have reached the patient several steps down the chain of events. The dosimetric severity scale could not fully be expressed by dose; it will be better if evaluated with biological effective dose. For a near miss reported in our study, in which the physician's prescription of 60.00 Gy/2.0 Gy/30 f was planned with 60.00 Gy/0.3 Gy/200 f, the dose was the same, but it would result in severe effect if not found.

33 near misses/incidents were reported in two years in this study. The volume of reports varies with the report criteria, quality and safety culture, equipment, and techniques used for different institutions. Mutic et al. have observed an incident report rate of 1 per 1.6 patients treated (this includes both incidents that reach the patient and near-miss incidents that are intercepted before reaching the patient) [16]. We collected a large number of incidents including near misses with very limited or zero clinical impact on patients, such as the plans of suboptimal plan quality, efficiency, beam energy, beam orientation, susceptibility to setup error, and organ motion, which were found and corrected prior to treatment. Such an approach facilitates continuous proactive improvement which can lead to the correction of small and/or latent system weaknesses before they result in much more severe events, to improve the safety and quality of care by supporting the systematic learning from errors [17, 18].

The rate of reported near misses/incidents decreased significantly in the second year, although more near misses of suboptimal plan quality were reported due to the enhanced quality and safety culture in the second year. The high rate of reported near misses/incidents in the first year may be attributed to the introduction of new equipment, new techniques, and new staff. Through the analysis of the occurrence of near misses/incidents, the cause/contribution factors, we found the weaknesses in the clinical process and implemented series of proactive measures to enhance the safety and quality. At the start of 2013, we introduced a more comprehensive check list in an attempt to reduce these errors and the error rate fell dramatically. Morganti et al. report a reduction in error through the use of independent checks [19]. Clear communication program et al. also contributed to the decrease, which included the efforts focusing on communication ambiguities (e.g., technicians not informed of cancelled treatment, changed plan or special appointment, or change in number of treatments, doctor unavailable for assigned appointment, etc.) and unclear physician directive, and additional policy and procedure changes addressing verbalization of treatment parameters prior to treatment delivery, laterality and documentation of change in prescription, and planning. Other examples of such interventions include changes in staffing levels to concentrate effort in more vulnerable parts of the process and modification of the quality assurance processes to focus on weaknesses in the treatment preparation process, enhance staff training, competency evaluation and supervision, strictly follow clinical protocol, and standardization of processes.

An important feature of the incident learning system is that it requires an investigation of sufficient depth to discover the basic causes of an incident according to a predetermined categorization and thereby facilitate the determination of meaningful corrective actions. An added benefit of such an approach is that it enables the identification of system problems or basic causes that could precipitate a range of different incidents. If the basic causes are addressed, it can be expected that overall system safety is enhanced and not just a particular weakness associated with a particular incident. The distribution of basic causes demonstrated that negligence and training contributed the most to the errors. It is understood for the new established program with new staff. The decrease of the number of these factors also demonstrated the effect of enhanced training and continuing education. Following that was policy not followed which was associated with a significant proportion of errors. The number of near misses/incidents assigned a basic cause relating to policy not followed was 12 and 3 for the first and second year, respectively. The reducing of these numbers indicated that the culture of following policies and procedures may be improving, which was also shown as the rising number of suboptimal plan quality near misses. There were still a significant number of near misses occurred due to failure to develop an effective plan, which showed the challenge of long term continuing education and personnel capacity improvement.

Systematic, scientific, efficient, and feasible management tool and approach is needed for the safety and quality management of radiotherapy. The aim of the incident learning was to capture of potential as well as actual incidents, engender a culture of safety, and support process improvement in patient care and safety. Incident learning can be used individually or combined with Failure Modes and Effects Analysis (FEMA), PDCA circle, and six sigma. Incident learning permits the implementation of both proactive and reactive error management initiatives, leading to quality improvement in all aspects of the clinic's operation and this strategy. A clear understanding of the potential consequences and relationships between different incident types will guide incident reporting, resource allocation, and risk management efforts. Additional work is needed to develop methods that can more effectively utilize reported data for process improvement.

## 5. Conclusions

Our results show that implementation of an effective incident learning system may serve to reduce the occurrence of actual incidents, enhance the culture of safety, and encourage the reporting of potential incidents as a proactive means of enhancing safety and quality in a radiation treatment program. Incident learning can be used for the safety and quality management of radiotherapy according to our primary experience.

## Figures and Tables

**Table 1:** Summary of reported incidents and near misses.

	Incident	Near miss	Patients treated	Incident (%)	Near miss (%)	Total (%)
First year	3	19	537	0.56	3.54	4.10
Second year	2	9	725	0.28	1.24	1.52

Total	5	28	1262	0.40	2.22	2.61

**Table 2:** Summary of reported incidents (in parentheses) and near misses category.

Category	First year	Second year	Total
Wrong patient	(1)	0	1

Wrong site	4 (2)	0 (1)	7
Wrong laterality	4	2	6
Wrong dose	4	1	5
Wrong prescription	5	2	7
Suboptimal plan quality	2	4 (1)	7

Total	19 (3)	9 (2)	33

**Table 3:** Summary of the origin of reported incidents (in parentheses) and near misses.

Category	First year	Second year	Total
Imaging for planning (simulation)	5	0	5

treatment planning	13 (1)	9 (2)	25
Plan transfer	(1)	0	(1)
Commissioning	1	0	1
Treatment delivery	(1)	0	(1)

Total	19 (3)	9 (2)	28 (5)

**Table 4:** Dosimetric severity distribution of reported incidents (in parentheses) and near misses.

Category	First year	Second year	Total
1/2	5 (3)	5 (2)	10 (5)
3/4	2	1	3
5/6	3	3	6
7/8	3	0	3
9/10	6	0	6

Total	19 (3)	9 (2)	28 (5)

**Table 5:** Medical severity distribution of reported incidents (in parentheses) and near misses.

Category	First year	Second year	Total
0	(1)	0	(1)
1	7 (2)	6 (2)	13 (4)
2	1	0	1
8/9	9	3	12
10	2	0	2

Total	19 (3)	9 (2)	28 (5)

**Table 6:** Summary of causes/contributory factors for reported incidents (in parentheses) and near misses.

Category		First year	Second year	Total
1biv	policy not followed	7 (5)	3	10 (5)

1cii	Appropriate skills not acquired from vendor provided training	2 (1)	1	3 (1)

1ciii	inadequate periodic assessment of staff competencies	3	1	4
1civ	lack of continuing education	3	1	4
1div	Communication	2	1	3
2bii	equipment design and construction issues	1	0	1
2biv	networking problems	(1)	0	(1)
3f,	Negligence	12 (3)	4	16 (3)
6aiii	failure to detect a developing problem	1	0	1
6div	failure to develop an effective plan	1	4	5
6eii	failure to execute the planned action	(1)	0	(1)
7	Other	0	2	2

Total		32 (11)	17	49 (11)

Comments: The data indicate the number of causes/contributory factors, not the number of near misses/incidents.
